# Interview Invitations for Otolaryngology Residency Positions Across Demographic Groups Following Implementation of Preference Signaling

**DOI:** 10.1001/jamanetworkopen.2023.1922

**Published:** 2023-03-07

**Authors:** Steven D. Pletcher, C. W. David Chang, Marc C. Thorne, Sonya Malekzadeh, Carrie L. Francis, Bobby Naemi, Joseph Costa, Dana Dunleavy

**Affiliations:** 1 Department of Otolaryngology–Head and Neck Surgery, University of California, San Francisco; 2 Department of Otolaryngology–Head and Neck Surgery, University of Missouri, Columbia; 3 Department of Otolaryngology–Head and Neck Surgery, University of Michigan, Ann Arbor; 4 Department of Otolaryngology–Head and Neck Surgery, MedStar Georgetown University, Washington, District of Columbia; 5 Department of Otolaryngology–Head & Neck Surgery, University of Kansas Medical Center, Kansas City; 6 Admissions and Selection Research and Development, Association of American Medical Colleges, Washington, District of Columbia

## Abstract

**Importance:**

Preference signaling is a new initiative in the residency application process that has been adopted by 17 specialties that include more than 80% of applicants in the 2023 National Resident Matching cycle. The association of signals with interview selection rate across applicant demographics has not been fully examined.

**Objective:**

To assess the validity of survey-based data on the association of preference signals with interview offers and describe the variation across demographic groups.

**Design, Setting, and Participants:**

This cross-sectional study examined the interview selection outcomes across demographic groups for applications with and without signals in the 2021 Otolaryngology National Resident Matching cycle. Data were obtained from a post-hoc collaboration between the Association of American Medical Colleges and the Otolaryngology Program Directors Organization evaluating the first preference signaling program used in residency application. Participants included otolaryngology residency applicants in the 2021 application cycle. Data were analyzed from June to July 2022.

**Exposures:**

Applicants were provided the option of submitting 5 signals to otolaryngology residency programs to indicate specific interest. Signals were used by programs when selecting candidates to interview.

**Main Outcomes and Measures:**

The main outcome of interest was the association of signaling with interview selection. A series of logistic regression analyses were conducted at the individual program level. Each program within the 3 program cohorts (overall, gender, and URM status) was evaluated with 2 models.

**Results:**

Of 636 otolaryngology applicants, 548 (86%) participated in preference signaling, including 337 men (61%) and 85 applicants (16%) who identified as underrepresented in medicine, including American Indian or Alaska Native; Black or African American; Hispanic, Latino, or of Spanish origin; or Native Hawaiian or other Pacific Islander. The median interview selection rate for applications with a signal (48% [95% CI, 27%-68%]) was significantly higher than for applications without a signal (10% [95% CI, 7%-13%]). No difference was observed in median interview selection rates with or without signals when comparing male (46% [95% CI, 24%-71%] vs 7% [95% CI, 5%-12%]) and female (50% [95% CI, 20%-80%] vs 12% [95% CI, 8%-18%]) applicants or when comparing applicants who identified as URM (53% [95% CI, 16%-88%] vs 15% [95% CI, 8%-26%]) with those who did not identify as URM (49% [95% CI, 32%-68%] vs 8% [95% CI, 5%-12%]).

**Conclusions and Relevance:**

In this cross-sectional study of otolaryngology residency applicants, preference signaling was associated with an increased likelihood of applicants being selected for interview by signaled programs. This correlation was robust and present across the demographic categories of gender and self-identification as URM. Future research should explore the associations of signaling across a broad range of specialties and the associations of signals with inclusion and position on rank order lists and match outcomes.

## Key Points

**Question:**

Are preference signals associated with interview selection rate for otolaryngology residency applicants across demographic groups?

**Findings:**

This cross-sectional study of 636 otolaryngology applicants found that applicants had a nearly 5-fold greater likelihood of being selected for interview at signaled programs than applicants who did not signal. This association was similar regardless of gender and self-identification as underrepresented in medicine.

**Meaning:**

These findings suggest that preference signaling, which has now expanded to include more than 80% of residency applicants across 17 specialties, was not associated with disadvantaging women or applicants who identify as underrepresented in medicine.

## Teaser

This cross-sectional study assesses the validity of survey-based data on the association of preference signals with interview offers and describes how the association varies across demographic groups.

## Introduction

During the residency application process, most otolaryngology–head and neck surgery (OHNS) applicants are eliminated from consideration during the interview selection phase. Prior to the initiation of preference signaling, there was no formal process to consider applicant preferences while programs were making interview selection decisions. The challenge of aligning applicant and program interests during the interview selection phase has been exacerbated by a surge of applications. Within OHNS, students submitted a mean of 84 residency applications in the 2022 National Resident Matching cycle, a 25% increase over the past 5 years. Furthermore, the number of OHNS applicants has increased, resulting in a doubling of applications received by programs.^1^

This increase in applications challenges the ability of programs to select from hundreds of applicants and may result in programs relying on algorithms and numerical screening metrics, including US Medical Licensing Examination (USMLE) scores. USMLE Step 1 is a licensure examination and scores are neither designed for use in selection decisions nor associated with residency performance.^2^ Within OHNS, an overemphasis on USMLE scores may result in disproportionately low recruitment of applicants who identify as women and as underrepresented in medicine (URM), defined as American Indian or Alaska Native; Black or African American; Hispanic, Latino, or of Spanish origin; or Native Hawaiian or other Pacific Islander.^3^ Residency application review also occurs in an environment of informal signaling, with the potential to exacerbate inequities.^4^ Applicants have differential access to mentors who advocate on their behalf or guide them through effective avenues for expressing interest prior to interview selection. In the absence of formal signals, residency selection committees may infer applicant preference based on perceived geographic ties, prior training institutions, or other factors subject to the bias of the committee.

Preference signaling was implemented in OHNS^5^ with the goals of mitigating a surge in applications,^1^ aligning program and applicant interests during the interview selection phase, and enhancing the capacity for holistic review, the preferred method for candidate assessment.^6,7^ While preference signaling has not previously been used in the residency application process, this system was developed and implemented in the economics PhD marketplace^8^ and several authors^9,10,11,12^ have advocated for this approach during the residency selection process.

Preference signaling in OHNS was evaluated with surveys sent to program directors and OHNS applicants in the 2021 National Resident Matching cycle demonstrating a significant association between preference signals and interview selection rate. Additionally, signaling was found to be popular among both applicants and program directors.^13^ However, these data are limited by survey response rates of 42% for applicants and 52% for programs.

Following this initial experience, the use of preference signals during the residency application process has expanded greatly: in the 2022 National Resident Matching cycle, the Association of American Medical Colleges (AAMC) and Electronic Residency Application Service (ERAS) offered preference signaling through a supplemental application for 3 specialties (general surgery, dermatology, and internal medicine) and now offers this service to 15 specialties in the 2023 National Resident Matching program.^14^ Urology adopted preference signaling in the 2022 National Resident Matching cycle and, along with OHNS, continues this program independent of AAMC and ERAS. With 17 specialties participating in preference signaling, more than 80% of residency applicants are anticipated to apply to specialties that use preference signaling.

New initiatives with uncertain outcomes across demographic groups must be evaluated to prevent exacerbation of existing disparities and ideally will contribute to reducing disparities. This is particularly important for OHNS where, despite an increase in medical school matriculation for women and students identifying as URM,^15^ the workforce lacks gender and ethnic diversity within residency programs and among practicing physicians.^16,17,18^

Widespread adoption of preference signaling necessitates a deeper assessment of this system which includes the results of signaling across demographic groups. The goal of this study is to validate the survey-based data on the association between signals and interview offer rate and to understand how this association varies across demographic groups and USMLE Step 1 scores. Although Step 1 has moved to pass or fail, inclusion of this metric provides a historical record of how scores were used and may help inform approaches for future residency selection cycles.

## Methods

This cross-sectional study was approved by the American Institutes for Research Review of Safeguards for Human Subjects. This study reported aggregate deidentified results obtained; therefore, no consent was required by the institutional review board. The report follows the Strengthening the Reporting of Observational Studies in Epidemiology (STROBE) reporting guideline.

During the 2021 National Resident Matching cycle, OHNS applicants were provided 5 signals to send to programs of particular interest. A website platform was created by the Otolaryngology Program Directors Organization council (OPDO)^19^ to disseminate guidance to applicants, provide best practice recommendations to programs, and collect signal submissions. Website creation, signal collection, and signal distribution were performed by existing OPDO staff; participation in signaling was free for both applicants and programs. Applicants were instructed not to signal their home program nor a program where they completed an in-person away rotation within the current academic cycle.

In the inaugural year of the OHNS signaling program, 100% of residency tracks (125 programs) participated in signaling. Some programs with multiple tracks (eg, research, clinical) chose to have a single signaling option for their institution while others requested a separate signaling opportunity for each track. For research purposes, research track signals and clinical track signals to the same institution were counted toward the parent program aggregately, resulting in 118 programs in the study, a 100% participation rate. Of 636 OHNS applicants in 2021, 548 unique applicants (86%) participated in signaling.

Preference signal data was linked to ERAS data using the applicants’ AAMC ID number. The association between preference signals and the likelihood of being selected for interview was analyzed for the entire applicant cohort as well as for gender and self-identified URM status. URM was defined as applicants who self-identified as 1 or more of the following racial and ethnic categories: American Indian or Alaska Native; Black or African American; Hispanic, Latino, or of Spanish origin; or Native Hawaiian or other Pacific Islander. Applicants were divided into terciles based on their most recent USMLE Step 1 score.

**Figure 1.**
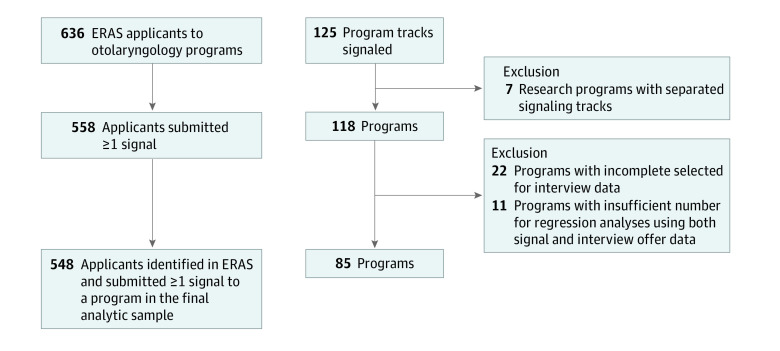
Flowchart of Data Selection A total of 636 applicants applied to 118 otolaryngology programs, resulting in 45 973 applications. The analysis sample was reduced to 548 applicants (87%) who participated in the signaling program. The final data set used for analyses represents 548 applicants (87%) and 85 programs (71%). ERAS indicates Electronic Residency Application System.

Because preference signals are designed to improve the interview selection process, interview selection rate was chosen as the primary outcome measure. These data were obtained from the ERAS Program Directors Workstation (PDWS), which contains a “selected for interview” status as an optional metric within its application tracking parameters. Many programs use methods other than ERAS to invite applicants for interviews; programs with incomplete selected for interview data were excluded. Based on feedback from OPDO members, programs with incomplete interview selection data were defined as those that designated fewer than 7 applicants per available match position as selected for interview. Removal of programs with absent or incomplete selected for interview data resulting in a final sample of 85 programs for analysis (Figure 1). Applications to home programs have a very high rate of resulting in an interview selection and were removed from this analysis to prevent artificial inflation of the interview selection rate to nonsignaled programs.

### Statistical Analysis

We conducted a series of logistic regression analyses at the individual program level. Analyses were conducted separately for each program and by applicant group because programs differ in how signals were incorporated into their selection process and the characteristics of each applicant group may differ. To complete a regression analysis, programs need adequate distribution of signal and nonsignal applications, as well as adequate numbers of women or URM applicants for these analyses. Programs that lack sufficient data for regression analysis comparing gender or URM status were excluded, resulting in 3 distinct samples to analyze this association with respect to gender, URM status, and for the entire cohort. These 3 program cohorts were largely representative of the OHNS programs overall, although the analytic sample programs received more applications, somewhat overrepresent programs in the highest mean USMLE Step 1 score range, and have an overrepresentation of larger programs (eTable in Supplement 1).

Each program within the 3 program cohorts (overall, gender, and URM status) was evaluated with 2 models. Model 1 explored the association between applicants’ signal status and interview invitation status. Signal status (coded as 0, indicating did not send a signal to program, and 1, sent signal to program) and interview selection (coded as 0, indicating did not receive an interview selection from program, and 1, received an interview selection from program) were treated as binary variables. Model 2 explored the association between signal status and interview invitation status while accounting for most recent USMLE Step 1 score. For the regression analyses in model 2, USMLE Step 1 scores were treated as a continuous covariate. However, for simplicity of presentation, probability results are displayed for 3 USMLE Step 1 score tercile categories, with each tercile corresponding to a range of scores that divides the applicant pool into the bottom, middle, and top third of scores.

Results were aggregated across programs by computing median probability of being selected for interview and the median 95% CIs across programs. The SD and minimum and maximum estimated probability also are reported. *P* values were 2-sided, and statistical significance was set at *P* = .05. Analyses were conducted using R version 4.1.3 (R Project for Statistical Computing). Data were analyzed from June to July 2022.

## Results

Of 636 US OHNS applicants, 548 (86%) participated in signaling, including 337 men (61%) and 86 applicants who identified as URM (16%) (Table 1). The mean, median, SD, and range for key variables in the overall analytic sample are summarized in Table 2. US MD applicants were more likely to participate in signaling (93%) than International Medical Graduates (46%) or DO applicants (59%).

**Table 1.** Demographic Characteristics of Applicants in the Full Sample

Characteristic	No. (%) (N = 548)
Gender	
Women	211 (39)
Men	337 (61)
URM status^a^	
Non-URM	374 (68)
URM	85 (16)
Unknown	89 (16)
Student type	
MD	485 (89)
DO	35 (6)
IMG	28 (5)

Abbreviations: IMG, international medical graduate; URM, underrepresented in medicine.

^a^ URM status is calculated by the applicant’s self-report race and ethnicity information and includes individuals who identified as American Indian or Alaska Native; Black or African American; Hispanic, Latino, or of Spanish origin; or Native Hawaiian or other Pacific Islander. Applicants were not required to provide race and ethnicity data on their application.

**Table 2.** Characteristics for the Full Sample of Programs (N = 85)

Characteristic	No.	
	Mean (SD)	Median (range)
Applications received	376.3 (94.5)	403 (89-498)
Signals received	27.0 (18.3)	22 (2-74)
Applicants selected for interview	49.5 (16.6)	49 (9-101)
USMLE Step 1 scores	244.4 (14.2)	247 (185-273)
Applicant URM status^a^		
Non-URM	265.3 (74.3)	288 (30-350)
URM	58.8 (15.4)	63 (16-78)
Applicant gender		
Women	145.2 (38.5)	158 (32-198)
Men	231.2 (56.5)	246 (57-300)

Abbreviations: URM, underrepresented in medicine; USMLE, United States Medical Licensing Examination.

^a^ Underrepresented in medicine status is calculated by the applicant’s self-report race and ethnicity information and includes individuals who identified as American Indian or Alaska Native; Black or African American; Hispanic, Latino, or of Spanish origin; or Native Hawaiian or other Pacific Islander. Applicants were not required to provide race and ethnicity data on their application.

Participating programs received a mean (SD) 376.3 (94.5) applications and 27.0 (18.3) signals and offered 49.5 (16.6) interviews (Table 2). The distribution of signals was skewed, with 25% of programs receiving 50% of signals.^13^ The mean number of applications submitted by otolaryngology applicants increased in the first year of signaling, from 68.8 in the 2020 Match cycle to 72.8 in the 2021 Match cycle.^20^

The selected to interview invitation rate was low among participating programs (median [range], 13% [4%-30%] of applicants; mean [SD], 13% [4.3%] of applicants). Of 548 participants in the study sample, 29 were not selected for interview by any of the 85 programs in the study sample.

### Preference Signals and Interview Invitations

**Figure 2.**
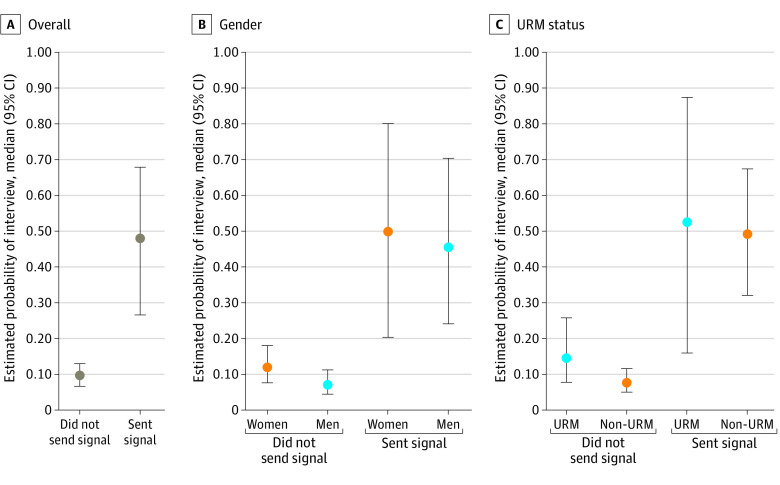
Probability of Interview Invitation With Signal Status Overall and by Gender and Underrepresentation in Medicine (URM) Status

Applications with a signal were significantly more likely to be selected for interview than nonsignal applications (48% [95% CI, 27%-68%] vs 10% [95% CI, 7%-13%]; *P* < .01) (Figure 2A). An increased interview selection rate associated with signals was found across gender (Figure 2B) and self-reported URM status (Figure 2C). There were no statistically significant differences between the median interview selection rates with or without signals when comparing male (46% [95% CI, 24%-71%] vs 7% [95% CI, 5%-12%]) and female (50% [95% CI, 20%-80%] vs 12% [95% CI, 8%-18%]) applicants. Interview selection rates for signaling URM applicants (53% [95% CI, 16%-88%]) were similar to those for non-URM signaling applicants (49% [95% CI, 32%-68%]). Furthermore, interview rates for nonsignaling URM applicants (15% [95% CI, 8%-26%]) were also similar to rates among nonsignaling non-URM applicants (8% [95% CI, 5%-12%]). There was considerable variability in the association of signals and selection for interview between programs (mean, 47.9%; SD, 19%; range, 11%-92%).

### Preference Signals and Interview Offer Rate Across Demographic Groups, Stratified by USMLE Score

**Figure 3.**
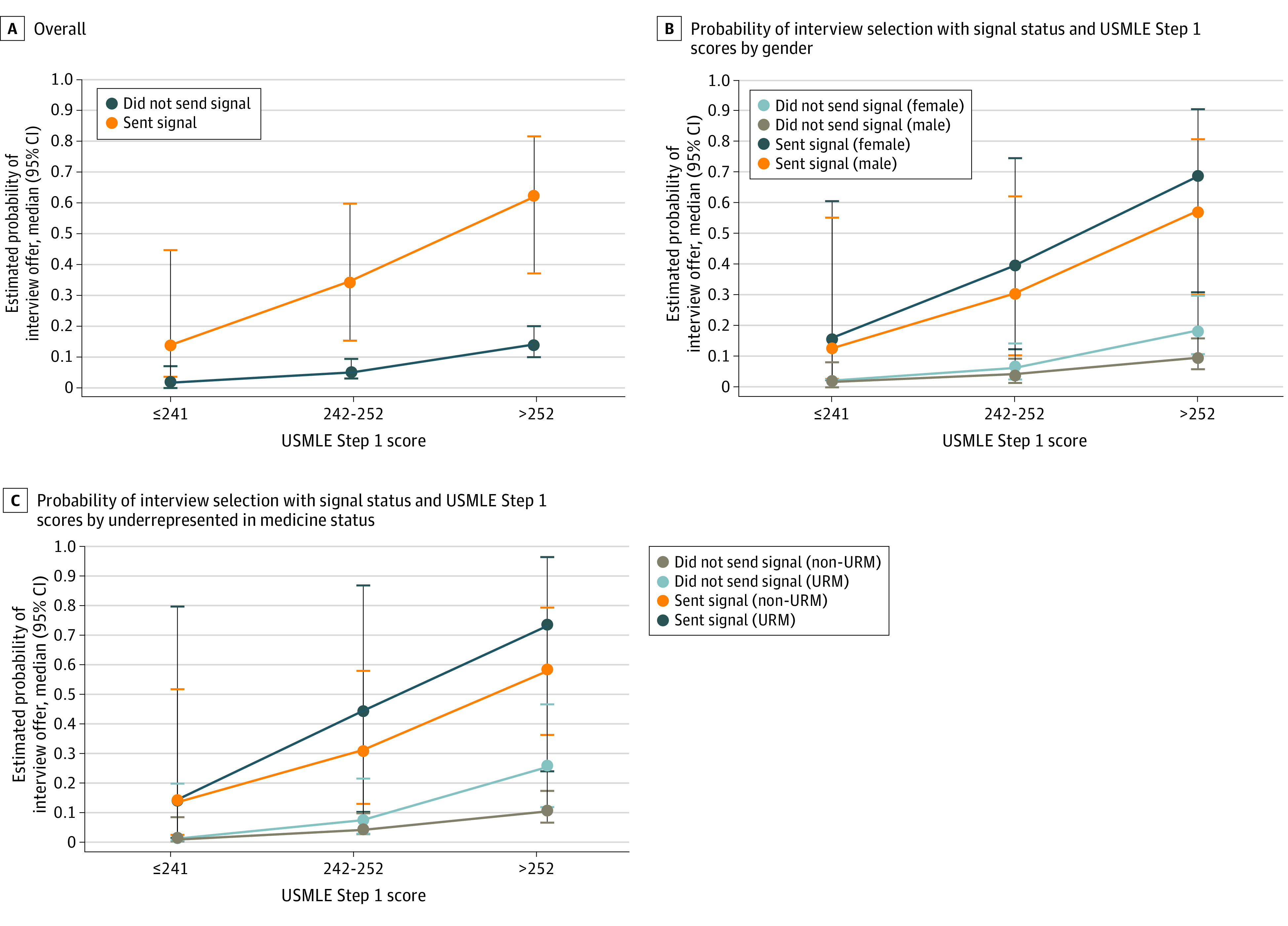
Probability of Interview Invitation With Signal Status and US Medical Licensing Examination (USMLE) Step 1 Scores Overall and by Gender and Underrepresented in Medicine (URM) Status

Signals were associated with a marked increase in the likelihood of being selected for interview across all groups and USMLE Step 1 score categories (Figure 3). Applications with a signal and a bottom tercile USMLE Step 1 score had the same likelihood of being selected for interview (14%) as the top tercile of USMLE Step 1 applications without a signal. Women applicants and applicants identifying as URM in the top tercile of USMLE Step 1 scores had the highest likelihood of receiving an interview selection (Figures 3B and C), but interview selection rates were not statistically different from those of men and non-URM applicants.

## Discussion

This cross-sectional study evaluates interview selection rates with respect to signals, applicant demographics, and USMLE Step 1 scores. Consistent with the self-reported applicant survey data,^13^ signals were associated with increased likelihood of being selected for interview. Along with validating survey-based data demonstrating this correlation, this study found that the positive association between signaling and interview selection rate held across demographic groups. There were no statistically significant differences observed in the interview offer rates associated with signal or nonsignal applications across gender or self-reported URM status. Our study found lower participation rates in the signaling program by applicants from osteopathic schools and international medical graduates.

USMLE scores were also positively associated with interview selection rate, and signals were associated with an increase in the interview selection rate across USMLE score ranges. USMLE scores are frequently used to screen applications during interview selection, a practice that may exacerbate the lack of diversity within OHNS residency programs.^3^ For an individual application, the presence of a signal may help mitigate the emphasis on USMLE scores in the interview selection process. Applications with a signal and a bottom tercile USMLE Step 1 score had the same likelihood of being selected for interview as the top tercile of USMLE Step 1 applications without a signal (14%).

One goal of signaling is to mitigate the challenges associated with increased application numbers. While signals were not associated with a decrease in the number of applications submitted per applicant, they do provide a tool that may simplify the application review process. For example, many programs report using signals as a tie-breaker when selecting students to invite for interviews.^21^ Future iterations of signaling with high signal numbers may impact application numbers. Implementation of a 30-signal program in orthopedic surgery was associated with an 11% decrease in the mean number of applications submitted per student.^20^

### Limitations

This study has several limitations. First, the study does not explore the association between signals and interview offers for many applicant types that may be considered URM, including first-generation applicants, low-income applicants, and specific racial or ethnic groups within the URM designation. Additionally, this study treats gender as binary and does not explore intersectional identities. Limited samples of these applicant types precluded such analysis in our study.

The data set for this analysis is incomplete. Not all programs entered interview selection information into ERAS. For those that did enter data into the selected for interview category, the completeness of these data is not known. To provide meaningful comparisons between demographic subgroups, we restricted our analyses to programs receiving an adequate number of signals from both men and women or both URM and non-URM applicants to complete a regression analysis. The relatively small proportion of applicants who identified as URM resulted in a smaller sample size for these evaluations and large CIs around the reported estimated probabilities. Therefore, it is important not to overinterpret small differences and to study the validity of results as more data become available.

We did not assess variability in how individual programs may interpret, use, or otherwise value signals. Although rates of concordance between signal and selection for interview status varied widely among programs, signals are an indication of applicant interest, not applicant congruence with program qualifications. Therefore, when programs decline to invite applicants who signal for an interview, our data provide no insight into whether this was due to a lack of signal value or a lack of program prioritization of an individual applicant.

The likelihood of being selected for interview by signaled programs is not only associated with the presence of a signal, but also with preexisting factors that drive the applicant’s interest: geography, alignment of clinical and research training and career goals, and department culture. In our 2022 survey-based signal analysis, these confounding factors were mitigated by identifying a comparable nonsignal program,^13^ ie, the program that the applicant would have signaled had they been provided with 1 additional signal. In this study, comparable nonsignal program data were not available, so interview selection rate may be confounded by potential differences in the programs that applicants selected to signal relative to those that they did not signal. Data from the OHNS signaling survey suggest only a modest increase in the interview selection rate for the comparable nonsignal program relative to selection rate overall for nonsignal programs (23% vs 14%) with a much higher interview selection rate for signal programs (58%).^13^

Data from this study may not be replicated in other specialties that implement preference signaling. OHNS is a small surgical subspecialty with a 63% match rate and no unmatched residency slots in the 2021 National Resident Matching cycle. These characteristics vary significantly from many of the programs that will be participating in preference signaling during the 2023 National Resident Matching cycle. Additionally, this is a retrospective study that makes use of data from previous admissions and selection cycles with different selection metrics available for evaluation. USMLE Step 1 scores were included to provide a context in the selection process at the time; inclusion of this data does not endorse use of USMLE scores for admissions and selection decisions by programs.

Applicants were instructed not to signal their home program or programs where they completed an in-person visiting rotation in the same academic year. Home programs were excluded from the nonsignal category, but it was not possible to identify which students completed an in-person visiting rotation, so these programs were included within the nonsignal group, likely artificially inflating the interview selection rate within this group. The potential impact from this is believed to be modest, as more than 80% of applicants identified a home program and were not eligible to complete a visiting rotation during this first year of the COVID-19 pandemic. Categorization of visiting rotation programs as nonsignal programs would bias the results of this study by decreasing the association between signals and selected for interview status; our study found a robust association despite this potential miscategorization.

## Conclusions

This cross-sectional study found that preference signals were associated with a higher likelihood of OHNS residency applicants being selected for interview by signaled programs. This association was robust and present across the demographic categories of gender and self-identified URM status.

Future signaling programs should provide educational outreach to international medical graduates and applicants from osteopathic schools. Additional research is needed to explore the effect of signaling across a broad range of specialties and on later-stage outcomes of the National Resident Matching cycle, including inclusion and position on rank order lists and match outcome.

## Notes

Supplement 1. **eTable.** Comparison of Program Characteristics for the Study Samples and All Otolaryngology Programs

Supplement 2.
Data Sharing Statement
