# Supplementary material for: Interview Invitations for Otolaryngology Residency Positions Across Demographic Groups Following Implementation of Preference Signaling
Source: JAMA Netw Open. 2023 Mar 7;6(3):e231922. doi: 10.1001/jamanetworkopen.2023.1922 (PMC9993176; doi:10.1001/jamanetworkopen.2023.1922)
Supplement: Supplement 1. — eTable. Comparison of Program Characteristics for the Study Samples and All Otolaryngology Programs [file jamanetwopen-e231922-s001.pdf]

Supplemental Online Content

Pletcher SD, Chang CWD, Thorne MC, et al. Interview invitations for otolaryngology residency positions across demographic groups following implementation of preference signaling. *JAMA Netw Open*. 2023;6(3):e231922. doi:10.1001/jamanetworkopen.2023.1922

eTable. Comparison of Program Characteristics for the Study Samples and All Otolaryngology Programs

This supplemental material has been provided by the authors to give readers additional information about their work.

© 2023 Pletcher SD et al. *JAMA Network Open.*

eTable. Comparison of Program Characteristics for the Study Samples and All Otolaryngology Programs

	**Model 1 Full Sample N Programs = 85**	**Model 2 Gender Sample N Programs = 67**	**Model 3 URM* Sample N Programs = 46**	**Otolaryngology Population N Programs = 118**
**Average Number of Applications Received: Mean (SD)**	376 (95)	397 (83)	372 (32)	370 (97)
**Average USMLE Step1: Score, Mean (SD)**	244.4 (14.2)	244.4 (14.2)	245 (13.8)	244.4 (8.4)
**Region**	**N (%)**	**N (%)**	**N (%)**	**N (%)**
Central	26 (31%)	19 (28%)	13 (28%)	34 (29%)
Northeast	25 (29%)	19 (28%)	13 (28%)	33 (28%)
Southern	20 (24%)	16 (24%)	9 (20%)	33 (28%)
Western	14 (17%)	13 (19%)	11 (24%)	18 (15%)
**Program Size**	**N (%)**	**N (%)**	**N (%)**	**N (%)**
Total Residents ≤ 10	25 (29%)	12 (18%)	5 (11%)	43 (36%)
Total Residents 11 – 15	27 (32%)	23 (34%)	15 (33%)	37 (31%)
Total Residents > 15	34 (40%)	32 (48%)	26 (57%)	38 (32%)

© 2023 Pletcher SD et al. *JAMA Network Open.*
