# Supplementary material for: Interview Invitations for Otolaryngology Residency Positions Across Demographic Groups Following Implementation of Preference Signaling
Source: JAMA Netw Open. 2023 Mar 7;6(3):e231922. doi: 10.1001/jamanetworkopen.2023.1922 (PMC9993176; doi:10.1001/jamanetworkopen.2023.1922)
Supplement: Supplement 2. — Data Sharing Statement [file jamanetwopen-e231922-s002.pdf]

Data Sharing Statement

Pletcher. Interview Invitations for Otolaryngology Residency Positions Across Demographic Groups Following Implementation of Preference Signaling. *JAMA Netw Open*. Published March 07, 2023. doi:10.1001/jamanetworkopen.2023.1922

Data
Data available: Yes
Data types: Data dictionary
How to access data: steven.pletcher@ucsf.edu
When available: With publication

Supporting Documents
Document types: None

Additional Information
Who can access the data: Data will be provided for researchers who desire to perform similar analyses
Types of analyses: Assessment of preference signaling outcomes
Mechanisms of data availability: Data dictionary will be provided after approval of the proposal by the primary author and a representative from the AAMC collaborating team
